# A Case of Acute Colonic Pseudo-Obstruction (Ogilvie's Syndrome) in a Nonsurgical Patient with Plasma Cell Leukemia

**DOI:** 10.1155/2022/6431248

**Published:** 2022-11-23

**Authors:** Akbar Hamid, Jacob Sabu, Omar Elhawary, Isha Puri, Moro Salifu, Man Oh, Mary Mallappallil

**Affiliations:** ^1^Division of Nephrology, Department of Medicine, SUNY Downstate Health Sciences University, Brooklyn, NY, USA; ^2^Division of Nephrology, Department of Medicine, Kings County Hospital Center, Brooklyn, NY, USA

## Abstract

Ogilvie's syndrome, also known as acute colonic pseudo-obstruction (ACPO), is a rare, nonobstructive dilation of the colon of unclear etiology. We present the case of a patient who presented with Ogilvie's syndrome and significant hypokalemia due to colonic loss despite repletion. This case report demonstrates the difficulty in diagnosis, treatment, and outcome.

## 1. Introduction

Ogilvie's syndrome refers to pathologic dilation of the colon without underlying mechanical obstruction, occurring primarily in patients with significant comorbidities. The diagnosis is based on clinical and radiologic grounds [1]. The incidence of the disease is frequently cited as approximately 100 cases per 100,000 hospital admissions per year, with a higher prevalence in men. The average age at presentation is approximately 60 years. Surgical patients are more likely to be diagnosed on postoperative days 3–5 [2].

## 2. Case Presentation

**Figure 1:**
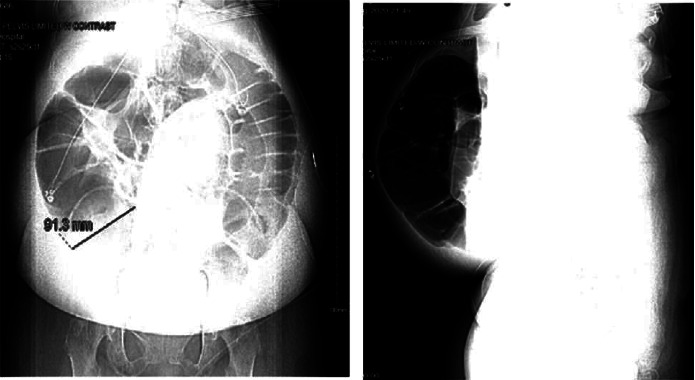
CAT scan with intravenous contrast showing dilated loops of bowel.

A 69-year-old African American woman with a history of type 2 diabetes mellitus, hypertension, hyperlipidemia, and elite control HIV infection (not on antivirals and with an undetectable viral load) presented with worsening lower back pain for a month. The laboratory tests showed marked leukocytosis, anemia, mild renal impairment, and a paraprotein gap. On skeletal survey, she was noted to have lytic lesions, and an MRI of the spine showed extensive compression, deformities, and epidural extension. A bone biopsy showed more than 80% blast cells with a marked increase in circulating plasma cells. She was diagnosed with plasma cell leukemia. In addition, she was also noted to have cauda equina syndrome. An abdominal CT scan showed a markedly dilated ascending colon, suggestive of obstruction, but she continued to have bowel movements. The patient successfully underwent chemotherapy induction and was discharged. When the patient was readmitted for the second cycle of chemotherapy (cisplatin and bortezomib), serum potassium was noted to be 1.8 mmol/L with a U wave on the electrocardiogram. However, serum magnesium levels were never below the normal range. She also complained of abdominal distension, diarrhea, and bilateral lower extremity edema. Multiple stool studies, including those for *Clostridioides difficile*, were negative. Several abdominal CT scans with intravenous contrast revealed dilated loops of the bowel without obstruction (Figure 1). She developed acute kidney injury, and the differential diagnosis included abdominal compartment syndrome; bladder pressure measured was noted to be elevated at 29 mmHg and decreased to 21 mmHg with the placement of a rectal tube. She became anuric, developed bacteremia with *Escherichia coli*, and subsequently died. See Table 1 for serum, urine, and stool electrolytes.

## 3. Discussion

A systematic review found that there was no uniform identification of ACPO in the literature, and inconsistent terminology prevented reliable data synthesis [3]. ACPO is a diagnosis of exclusion made by excluding infectious causes such as *Clostridium difficile*. Delays in diagnosis may result in ineffective supportive treatment and may risk unnecessary aggressive therapies. Our patient had findings consistent with ACPO on imaging and a negative gastrointestinal infectious workup.

While the exact pathophysiology is unclear, the current hypothesis suggests an imbalance of autonomic regulation in the distal colon [4]. Sympathetic innervation to the distal colon comes from T9–L2, whereas parasympathetic innervation is from S2–S4 sacral segments of the spinal cord [5]. Moreover, neurogenic bowel dysfunction can also result from medications, spinal trauma, and immobility. Our patient had morbidity that would increase systemic sympathetic expression due to the spinal cord and immobility.

Potassium homeostasis is maintained by the kidney and the gut. The big potassium (BK) channels, also called Maxi-K channels, are large conductance, voltage-gated, flow-mediated channels that are found in various tissues and widely expressed in the kidney and the distal intestinal colon crypts [6]. BK channels are associated with one of the four subunits (BK*β*1–*β*4) and are responsible for flow-induced K secretion [7].

Significant loss of fluid in diarrhea with hypokalemia is reported to be common [8]. The secretory diarrhea in ACPO is driven by potassium secretion, in contrast to the inhibition of sodium reabsorption or chloride secretion, which are the more common pathophysiologic mechanisms of other forms of secretory diarrhea [9]. It is common that patients may lose more than 100 millimoles of potassium daily. The source of hypokalemia appears to be gastrointestinal potassium loss. The kidney response to hypokalemia is compensatory potassium conservation with a low amount of potassium lost in urine. Our patient had an initial urine potassium of 23 mmol/L, and it peaked at 45.8 mmol/L; the first stool potassium was >100 mmol/L with a stool volume of 900 milliliters. A repeat stool study after a week showed stool potassium of 95.9 mmol/L and stool sodium of 42 mmol/L. Our patient received more than 200 mEq of potassium supplement a day, but her serum potassium level persistently remained below 3.5 mmol/L.

Imaging, including an abdominal x-ray on admission, showed distension of the colon, which measured up to 11.4 cm in diameter at the cecum and 8.2 cm at the transverse level. The difference in distension between the transverse and distal colon supports the autonomic dysfunction hypothesis since the splenic flexure is the point where innervation sources change [10]. Serial imaging of the bowel showed worsening diffused colonic dilation.

**Figure 2:**
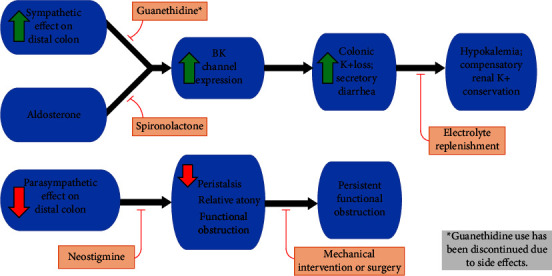
Pathophysiology and treatment options for acute colonic pseudo-obstruction.

Once a diagnosis is made, it is important to determine the pace of the treatment plan (Figure 2). Urgency in treatment includes a diameter of more than 12 cm, which is often considered less responsive to conservative therapy, and decompression may be needed to reduce the risk of colonic perforation [4].

If less pressing, conservative therapy with electrolyte replacement and pharmacologic therapy may be used. In an attempt to restore balance, historical studies on the treatment of ACPO have used the adrenergic blocker guanethidine, followed by neostigmine, which inhibits acetylcholinesterase and is a parasympathomimetic [11]. Questionable efficacy and side effects of postural hypotension led to discontinuation of the use of guanethidine. Neostigmine was tested successfully and is still prescribed in the management of ACPO [12]. As aldosterone upregulates the BK channels, a case report of aldosterone inhibition with spironolactone has also been tried with success [13].

Mechanical interventions may include rectal tubes, needle decompression, epidural anesthesia, percutaneous cecostomy, and various options for surgery. Surgery, which is usually considered as a final option, is associated with high rates of morbidity and mortality [14].

## 4. Conclusion

ACPO is a diagnosis of exclusion in patients with abdominal symptoms often accompanied by hypokalemia, which has high morbidity, but timely determination of the cause can prevent inadvertent surgery and complications.

## Figures and Tables

**Table 1:** Hypokalemia with significant stool loss of potassium and relatively low urine potassium.

Date	Serum potassium (mmol/L)	Urine osmolarity (mOsm/kg)	Urine potassium (mmol/L)	Urine sodium (mmol/L)	Urine chloride (mmol/L)	Urine magnesium (mg/dL)	Stool potassium (mmol/L)	Stool sodium (mmol/L)	Stool osmolarity (mOms/kg)
8/09/20	1.8								
8/11/20	2.6		23	<20	63		>100	42	
8/12/20	3.2	212	17	39					
8/15/20	2.7		18						
8/19/20	3.1		46	88	174				
8/20/20	3.4		45	37	134	10.4			
8/25/20	3.4		32	65	121		95.9	41	413
8/28/20	2.8		5	133	122			<20	
8/29/20	2.9		14	66	77				
9/1/20	3.8		14	110	75				

## Data Availability

The relevant data table is included in the case report.
